# REG4 promotes peritoneal metastasis of gastric cancer through GPR37

**DOI:** 10.18632/oncotarget.8442

**Published:** 2016-03-28

**Authors:** Hexiao Wang, Lei Hu, Mingde Zang, Baogui Zhang, Yantao Duan, Zhiyuan Fan, Jianfang Li, Liping Su, Min Yan, Zhenggang Zhu, Bingya Liu, Qiumeng Yang

**Affiliations:** ^1^ Shanghai Key Laboratory of Gastric Neoplasms, Department of Surgery, Shanghai Institute of Digestive Surgery, Ruijin Hospital, Shanghai Jiao Tong University School of Medicine, Shanghai 200025, People's Republic of China; ^2^ Affiliated Hospital of Jining Medical University, Department of Surgery, Jining 272000, People's Republic of China

**Keywords:** gastric cancer, peritoneal metastasis, REG4, adhesion, positive feedback

## Abstract

Being the major reason of recurrence and death after surgery, peritoneal metastasis of gastric cancer dooms the prognosis of advanced gastric cancer patients. Regenerating islet-derived family, member 4 (REG4) is believed to promote peritoneal metastasis, however, its mechanism is still a moot point at present. In the present study, we show that high expression of REG4 correlates with advanced stage and poor survival prognosis for gastric cancer patients. REG4 overexpression significantly enhances peritoneal metastasis by increasing adhesion ability. Moreover, SP1 is proved to be a transcription factor of REG4 and induce REG4 expression upon TGF-alpha stimulation. Also, G protein-coupled receptor 37 (GPR37) is identified to be in the same complex of REG4, which mediates REG4′s signal transduction and promotes peritoneal metastasis of gastric cancer cell. Interestingly, we also discover a positive feedback loop triggered by REG4, amplifying itself through EGFR transactivation, consisting of GPR37, ADAM17, TGF-alpha, EGFR, SP1 and REG4. In conclusion, REG4 promotes peritoneal metastasis of gastric cancer through GPR37 and triggers a positive feedback loop.

## INTRODUCTION

Gastric cancer is the third leading cause of cancer mortality, with more than 700, 000 deaths every year worldwide [1]. While we have witnessed a continuing progress in gastric cancer treatment [2], the average survival time of peritoneal dissemination patients is less than 6 months [3], thus, it is of great significance to investigate the mechanisms of peritoneal metastasis.

Regenerating islet-derived family, member 4(REG4) was originally isolated from a cDNA library of inflammatory bowel disease in 2001 [4]. It is a small secretive protein, whose function may be related to proliferation and regeneration in physiological conditions [5]. We have reported that REG4 is significantly over-expressed in gastric cancer tissues(especially in signet-ring cell carcinoma) than in corresponding normal tissues, and high expression of REG4 is positively related to lymph node metastasis [6]. Similarly, relevant studies showed that REG4 mRNA level in peritoneal washes of gastric cancer patients is closely associated with gastric wall penetration and that REG4 is an independent prognostic factor for peritoneal recurrence-free survival [7–9]. Although it has been reported that REG4 induces a series of anti-apoptosis gene expression through activating epidermal growth factor receptor (EGFR)/ protein kinase B (AKT)/ activator protein 1 (AP-1) signaling pathway [10], and that REG4 also increases mitogenesis involving AKT/ glycogen synthase kinase 3 beta (GSK3beta)/beta-Catenin/ transcription factor 4 (TCF-4) signaling in human colorectal cancer [11], no thorough investigation about the mechanism of peritoneal metastasis has been conducted, neither its receptor, nor its interactive partner has been identified for more than a decade.

The interesting part of REG4 is that it is able to activate EGFR pathway, in the meantime, REG4 mRNA can be induced by some EGFR ligands, [12] which suggests that a positive feedback loop of REG4 regulation is likely to exist in cancer tissues. While REG4′s interactive partner has not been identified so far, there are quite a few proofs that many G-protein coupled receptors are capable of transactivating EGFR through a disintegrin and metalloproteinase (ADAM) or matrix metalloproteinase (MMP) family, which cleaves the pro-ligands of EGFR [13–15]. In addition, several transcription factors downstream of EGFR are closely associated to poor outcome of gastric cancer patients [16, 17], which might contribute to REG4 inducement after EGFR ligands stimulation. As a result, such a self-amplifying circuit, through epigenetic modification, might exist in gastric cancer cells.

The present study shows that REG4 promotes peritoneal metastasis of gastric cancer through G-protein coupled receptor 37 (GPR37), and triggers a positive feedback loop.

## RESULTS

### REG4 expression in gastric cancer tissues

**Figure 1:**
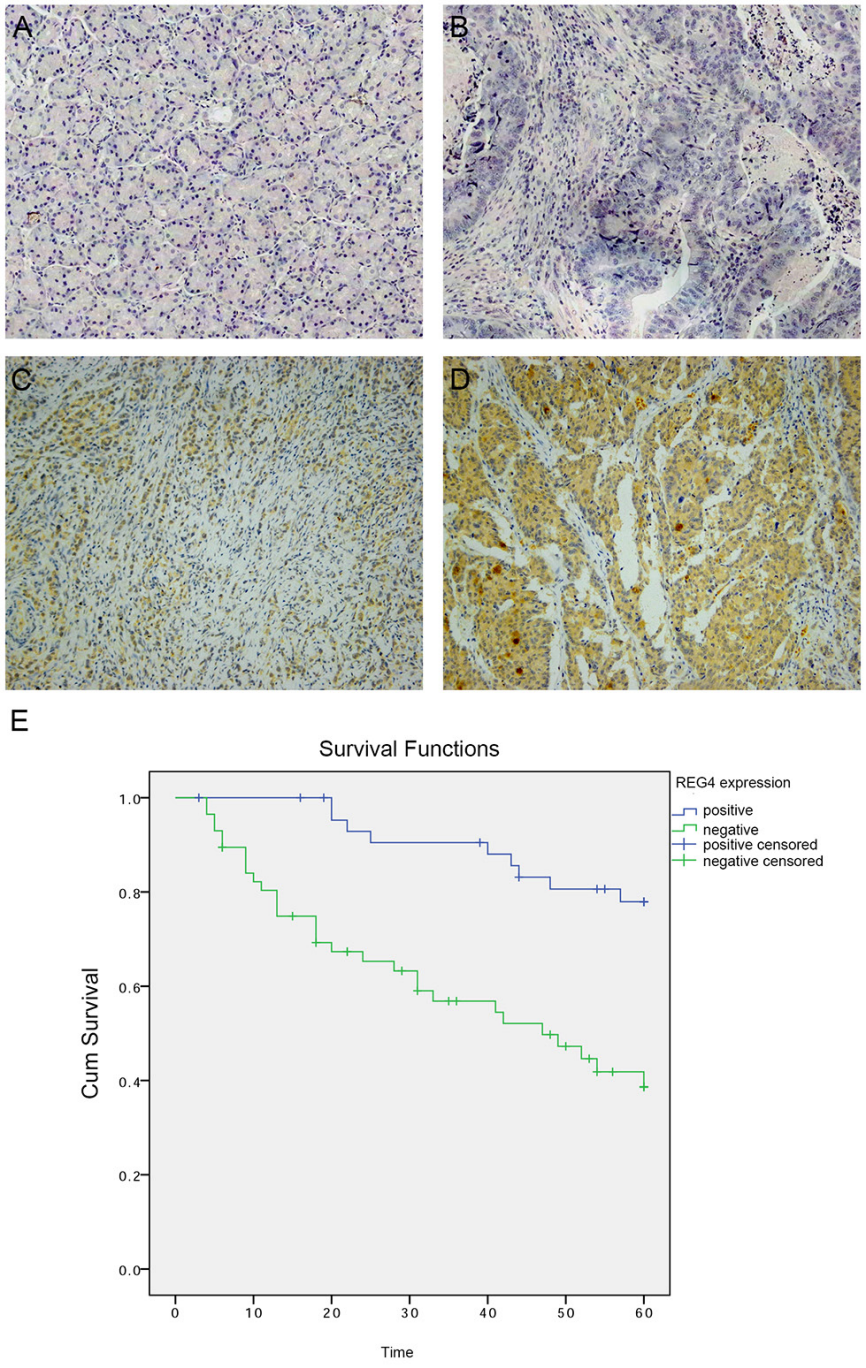
Expression of REG4 in clinical gastric cancer tissues **A.** Negative REG4 expression in non-tumor gastric mucosa. **B.** Negative REG4 expression in gastric cancer. **C.** Weak positive REG4 expression in gastric cancer. **D.** Strong positive REG4 expression in gastric cancer. **E.** Kaplan-Meier survival curve of patients positive and negative for REG4 expression.

To investigate REG4 expression pattern in gastric cancer, Immunohistochemistry was performed on paired tumorous and non-tumorous tissues isolated from 102 gastric cancer patients. It revealed that 57 out of 102 (55.9%) gastric cancer tissues were REG4 positive, predominantly located in the cytoplasm, however occasionally weak or no staining was observed in non-neoplastic tissues (p<0.01) (Figure 1A–1D). We next analyzed the correlation between REG4 expression and the clinicopathological status of gastric cancer patients. High REG4 expression was significantly associated with poor differentiation (p=0.004), lymph node metastasis(p=0.041), and advanced TNM stage(p=0.028), but was unrelated to other factors, such as, gender, age, tumor location, and T stage (Table 1).

**Table 1:** Association between Reg4 expression and clinicopathological factors of gastric cancer patients

Variables	Number (n=102)	Reg4 immunostaining		P
		Positive(n=57)	Negative(n=45)	
**Gender**				
**Male**	68	36	32	0.526
**Female**	34	21	13	
**Age(years)**				
**≥65**	35	21	14	0.675
**≥65**	67	36	31	
**Differentiation**				
**Well to moderate**	36	13	23	0.004
**Poor**	66	44	22	
**Tumor location**				
**Gastric fundus**	5	3	2	0.980
**Gastric corpus**	47	26	21	
**Pylorus**	50	28	22	
**T stage**				
**T1+T2**	77	42	35	0.652
**T3+T4**	25	15	10	
**Lymph node metastasis**				
**Negative**	40	17	23	0.041
**Positive**	62	40	22	
**Distant metastasis**				
**Negative**	97	52	45	0.065
**Positive**	5	5	0	
**TNM stage**				
**I+II**	60	28	32	0.028
**III+IV**	42	29	13	

Note: Positive Reg4 expression included all positive cases, such as weak and strong.

Kaplan-Meier survival curve with a median follow-up period of 49.5 months demonstrated that patients with low REG4 expression survive significantly longer than those with high REG4 expression(Figure 1E, p<0.01). High expression of REG4 was proved to be associated with a poor survival prognosis(hazard ratio=3.20, 95%CI, 1.41-7.25; p<0.01) by multivariate Cox regression analysis. These data indicate that high expression of REG4 correlates with advanced stage and is an independent risk factor for the survival of gastric cancer patients.

### REG4 promotes peritoneal metastasis of gastric cancer

**Figure 2:**
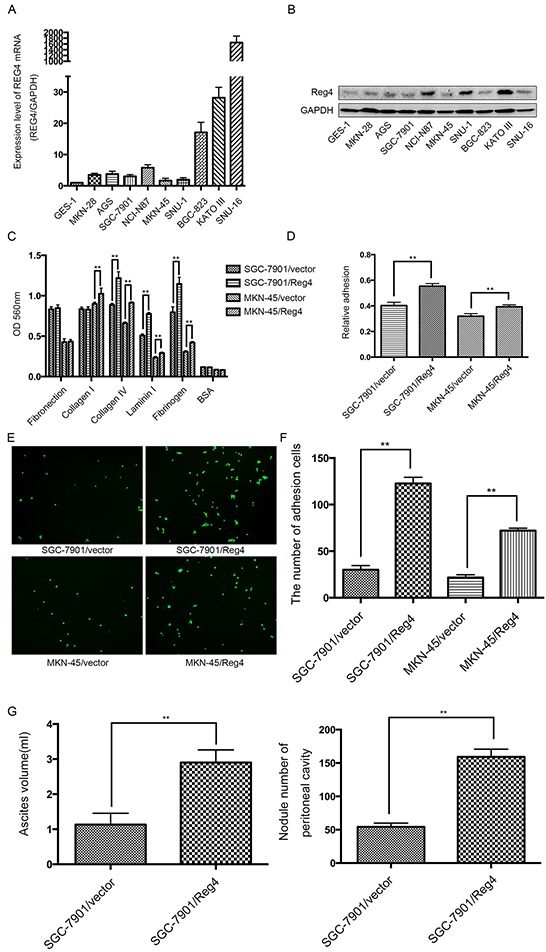
REG4 promotes adhesion and peritoneal metastasis of gastric cancer cells **A, B.** mRNA and protein level of REG4 were evaluated by qRT-PCR and Western Blot in 9 gastric cancer cell lines and 1 immortalized gastric epithelial cell line. **C, D.** Cells or percentage adherent to different ECM components or Matrigel coated plates after 30min incubation were quantified at OD 560nm. **E, F.** Representative images and number of cells adherent to murine peritoneum after 30min incubation. **G.** Ascites volume and disseminated tumor number collected in mice. Error bars correspond to mean ± SD of at least three independent experiments. *P < 0.05, **P<0.01.

We first examined REG4 expression in 9 gastric cancer celllines and 1 immortalized gastric epithelial cellline. qRT-PCR and Western blot showed that REG4, on both mRNA and protein level, was relatively more abundant in gastric cancer celllines and was dramatically upregulated in SNU-16 (Figure 2A–2B). Two stably transfected celllines were established, namely, SGC-7901/REG4 and MKN-45/REG4, verified by both qRT-PCR and Western blot (Supplementary Figure S1A-S1B).

Since adhesion to peritoneum is the prerequisite for peritoneal metastasis, we went on to evaluate the effect of REG4 on the adhesive behavior of gastric cancer cells by performing adhesion assays on plates coated with different extracellular matrix components. A dramatically increased adhesion to collagen IV, laminin I and fibrinogen was observed in SGC-7901/REG4 and MKN-45/REG4 cells (Figure 2C). To further corroborate the effect of REG4 on adhesive ability, adhesion assays using plates coated with Matrigel were performed, which, as expected, showed higher adhesion rates in REG4 overexpressed cells (Figure 2D). In addition, to simulate in vivo circumstances, we also examined adhesive ability to murine peritoneum, and such adhesion assays further confirmed that REG4, indeed, could enhance adhesive ability of gastric cancer cells (Figure 2E, 2F).

We next evaluated the in vivo effect of REG4 on peritoneal metastasis. Mice injected with REG4 overexpressed cells exhibited remarkable ascites formation ten weeks post-inoculation compared with that injected with control cells. Moreover, mice injected with REG4 overexpressed cells also developed tumors in the peritoneum, mesentery, and diaphragm, which exhibited more organ involvements in contrast with that injected with control cells (Figure 2G, Supplementary Figure S2A). Histological analysis of the xenografts confirmed the metastasis of peritoneum, mesentery and diaphragm (Supplementary Figure S2B). These in vitro and in vivo experiments suggest that REG4 promotes peritoneal metastasis of gastric cancer cells by increasing adhesion ability.

### GPR37 is in the same complex with REG4 and mediates its peritoneal metastasis ability

**Figure 3:**
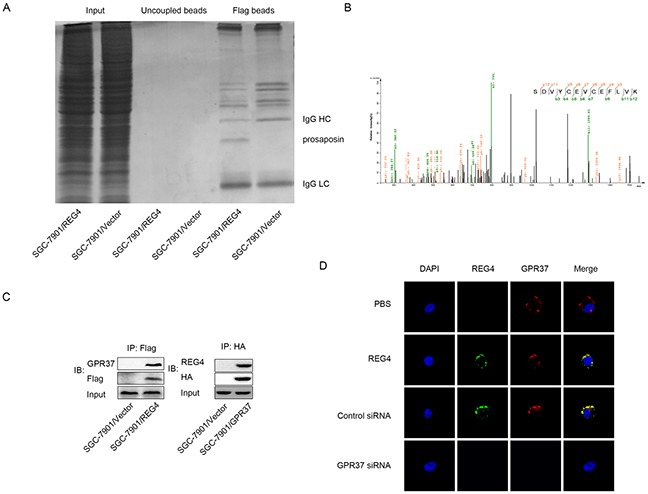
GPR37 is in the same complex with REG4 **A.** Immunoprecipitation of the whole cell extracts from SGC-7901/REG4-Flag and SGC-7901/Vector-Flag with anti-Flag antibody. **B.** Mass spectra results of the differential protein band in (A). **C.** Immunoprecipitation of the whole cell extracts from SGC-7901/REG4-Flag with anti-Flag antibody, and from SGC-7901/GPR37-HA with anti-HA antibody. **D.** Confocal microscopy for SGC-7901 cells stained with anti-REG4, anti-GPR37 antibody and iFluor 594 anti-rabbit IgG, Alexa488 anti-goat IgG second antibody. The cells were treated with PBS (row 1) or recombinant REG4 (rows 2–4), and were untransfected (row 2), transfected with control-siRNA (row 3), or GPR37-siRNA (rows4). Cell nuclei were counterstained with DAPI.

To identify the interactive partner, or even the receptor of REG4, the total protein of cells transfected with REG4-Flag plasmid and that of cells transfected with vector-Flag plasmid were immunoprecipitated with anti-Flag antibody, and resolved on a denaturing gel. A differential protein band was revealed after Coomassie blue staining and analyzed by liquid chromatography-mass spectrometry (Figure 3A). Unfortunately, we failed to identify any membrane protein in the band. However, another interesting small secretory molecule, prosaposin, was found in the band, whose receptor had just been proved to be a G-protein coupled receptor, G-protein coupled receptor 37 (GPR37) (Figure 3B). It was reported that prosaposin down-modulation decreases metastatic prostate cancer cell adhesion, migration, and invasion [18]. Since it is not uncommon for similarly functional molecules to coexist in the same complex or share one receptor to some degree, we, therefore, went on to evaluate this hypothesis.

Firstly, western blot was performed to verify GPR37 expression in gastric cancer cells (data not shown). Next, the interaction between GPR37 and REG4 was confirmed by immunoblotting of the immunoprecipitate with anti-GPR37 antibody and anti-REG4 antibody(Figure 3C, Supplementary Figure S3A). To further confirm whether REG4 colocalizes with GPR37 on gastric cancer cell membrane, confocal microscopy using different fluorescent antibodies for REG4 and GPR37 was performed, the image clearly demonstrated that REG4 colocalized with GPR37 on the cell membrane, and that knockdown of GRP37 prevented REG4 to associate with cell surface (Figure 3D, Supplementary Figure S3B).

**Figure 4:**
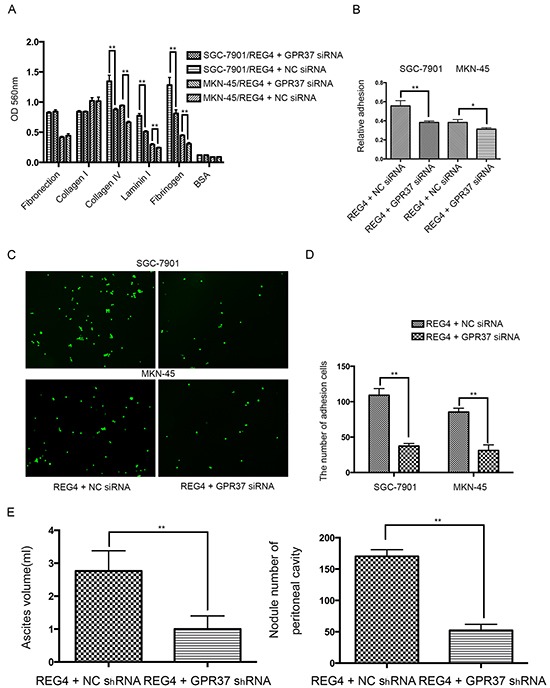
Knocking down GPR37 abrogates the pro-peritoneal metastasis effect of REG4 **A, B.** Cells or percentage adherent to different ECM components or Matrigel coated plates were quantified at OD 560nm after 30min incubation. **C, D.** Representative images and number of cells adherent to murine peritoneum after 30min incubation. **E.** Ascites volume and disseminated tumor number collected in mice. Error bars correspond to mean ± SD of at least three independent experiments. *P < 0.05, **P<0.01.

Then we knocked down GPR37 in REG4 overexpressed cell lines or rhREG4 stimulated cells to test their effects upon the biological behaviors of gastric cancer cells. As expected, GPR37 knockdown abrogated, to a great extent, the peritoneal metastasis abilities triggered by REG4. Adhesion abilities, either to extracellular matrix or murine peritoneum, were all significantly decreased (Figure 4A–4D) as well as peritoneal metastasis in nude mice (Figure 4E, Supplementary Figure S4A, S4B). These data strongly indicate that GPR37 is in the same complex with REG4, and mediates its signal transduction in peritoneal metastasis of gastric cancer.

### SP1 is the transcription factor of REG4 upon TGF-alpha stimulation

**Figure 5:**
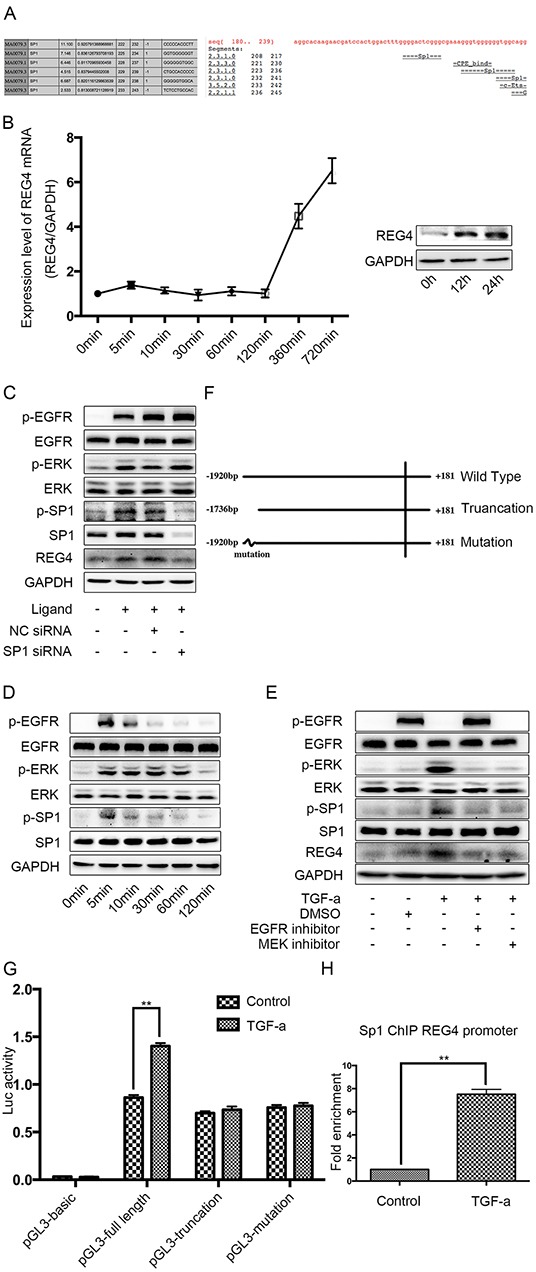
SP1 is the transcription factor of REG4 upon TGF-alpha stimulation in SGC-7901 **A.** Binding sites of SP1 on REG4 promoter were predicted by different software. **B.** TGF-alpha(10nM) increases REG4 on both mRNA and protein level. **C.** Knocking down SP1 abrogates REG4 inducement upon TGF-alpha stimulation. **D.** Phosphorylation level of EGFR, ERK, SP1 were analyzed after TGF-alpha(10nM) stimulation. **C, E.** Activation of EGFR pathway and expression of REG4 were tested with or without EGFR inhibitor, MEK inhibitor and SP1 siRNA, respectively, after TGF-alpha(10nM) stimulation. **F.** A brief scheme of wild type, truncation and mutation REG4 promoter cloned in pGL3-basic. **G.** Luciferase activity of a reporter construct harboring different REG4 promoter with or without TGF-alpha(10nM) stimulation. **H.** SP1 occupancy (fold enrichment) on REG4 promoter areas with or without TGF-alpha(10 nM) stimulation. Error bars correspond tomean ± SD of at least three independent experiments. *P < 0.05, **P<0.01.

It seems that REG4 expression is enhanced transcriptionally in gastric cancer cells, so we scanned the REG4 promoter for potential transcription factors. As a result, we found several SP1 binding sites on the promoter, one of them with a pretty high score (Figure 5A). Being a transcription factor downstream of epidermal growth factor receptor (EGFR), SP1 is significantly phosphorylated when the pathway is activated, which greatly increases its transcriptional function [19–21]. Therefore we used an EGFR ligand, TGF-alpha, to activate EGFR/extracellular regulated MAP kinase (ERK)/SP1 signaling pathway and detected REG4 expression. After TGF-alpha stimulation, a significant increase of both REG4 mRNA and REG4 protein was observed (Figure 5B, Supplementary Figure S5A) and siRNA mediated SP1 knocking down successfully abrogated REG4 inducement (Figure 5C, Supplementary Figure S5B). In order to prove the existence of this pathway, we performed western blot to test total and phosphorylated levels of EGFR, ERK and SP1 as well as protein level of REG4 by using EGFR inhibitor, MEK inhibitor and SP1 siRNA. The results showed that TGF-alpha activated EGFR, ERK, SP1 sequentially and finally promoted REG4 expression (Figure 5C–5E, Supplementary Figure S3B-S3D). Next, we corroborated the direct involvement of SP1 as a transcription factor in REG4 regulation by using Chromatin Immunoprecipitation (ChIP) and luciferase reporter assay (Figure 5F–5H, Supplementary Figure S5E, S5F).

**Figure 6:**
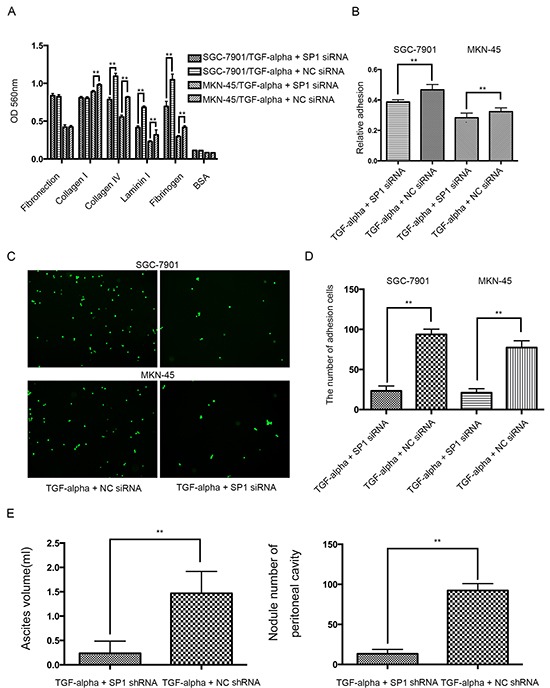
Knocking down SP1 abrogates the pro-peritoneal metastasis effect of REG4 **A, B.** Cells or percentage adherent to different ECM components or Matrigel coated plates were quantified at OD 560nm after 30min incubation. **C, D.** Representative images and number of cells adherent to murine peritoneum after 30min incubation. **E.** Ascites volume and disseminated tumor number collected in mice. Error bars correspond to mean ± SD of at least three independent experiments. *P < 0.05, **P<0.01.

To further substantiate the transcriptional effect of SP1 on REG4 after TGF-alpha stimulation, adhesion assays were performed using SP1 knocked down cell lines. Knocking down SP1 decreases adhesion ability to extracellular matrix, Matrigel and murine peritoneum after TGF-alpha stimulation (Figure 6A–6D). Moreover, mice injected with SP1 knocked down cell lines also developed less ascites and metastatic nodules. (Figure 6E, Supplementary Figure S6A, S6B). The data aforementioned suggest that,activated by TGF-alpha,SP1 induces REG4 expression as a transcription factor and promotes peritoneal metastasis of gastric cancer.

### REG4 triggers a positive feedback loop

Since GPCRs are able to transactivate EGFR through pro-ligand shedding, mediated by ADAM and MMP family [13–15], and, on the other hand, TGF-alpha, an EGFR ligand, is able to induce REG4 expression through SP1 activation. It seems that a positive feedback loop may exist in gastric cancer cells.

**Figure 7:**
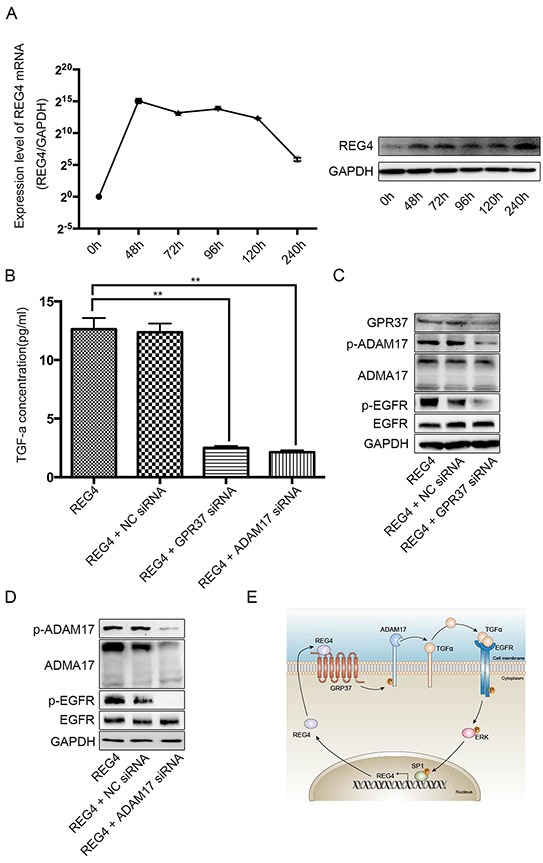
REG4 triggers a positive feedback loop **A.** mRNA and protein level after transient transfection of REG4 plasmid. **B.** TGF-alpha concentration in medium supernatant with or without GPR37 siRNA or ADAM17 siRNA after rhREG4 (10ng/ml) stimulation. **C, D.** Phosphorylation and total level of ADAM17 and EGFR with or without GPR37 siRNA or ADAM17 siRNA after rhREG4(10ng/ml) stimulation. **E.** A brief scheme of the positive feedback loop triggered by REG4.

In order to investigate such hypothesis, first of all, we transiently transfected cell lines with REG4 plasmid or treated with rhREG4, and, interestingly, the expression of REG4 was able to sustain a long time at a relatively high level, which indicates its self-sustaining property (Figure 7A, Supplementary Figure 7A, 7B). Next, to test whether GPR37 is able to promote EGFR ligands shedding like many other members of the G-protein coupled receptor family, we performed ELISA in cells stimulated by rhREG4 with or without GPR37 siRNA, and the results showed that the concentration of TGF-alphain the supernatant of cell culture medium was dramatically lower in cells transfected with GPR37 siRNA than that in cells with control vectors (Figure 7B, Supplementary Figure S7C). Since ADAM17 is one of the sheddases capable of cleaving transmembrane inactive pro-active TGF-alpha and releasing mature TGF-alpha into the medium [22], we went on to investigate if ADMA17 is involved in the process of TGF-alpha shedding. However, in contrast to some other stimulants [23], western blot did not reveal a significant change in either mature or immature form of ADAM17(Figure 7C, Supplementary Figure S7D). But siRNA mediated ADAM17 knocked down did remarkably prohibit TGF-alpha shedding and EGFR activation upon rhREG4 stimulation, which verified the upstream position and important effect of ADMA17(Figure 7B, 7D, Supplementary Figure S7C, S7E). As a result, we wondered if it was post-translational modification that did the work. Finally, western blot showed that phosphorylation of ADAM17 was indeed decreased after knocking down GPR37 upon rhREG4 stimulation(Figure 7C, Supplementary Figure S7D). Taken together, we proposed that REG4 is able to trigger a positive feedback loop: (1) REG4 activates GPR37, leading to ADAM17 phosphorylation, which cleaves the pro-TGF-alpha; (2) TGF-alpha activates EGFR/ERK/SP1 signaling pathway, and SP1 induces REG4 expression (Figure 7E).

## DISCUSSION

In the present study, we demonstrate that REG4 is able to promote peritoneal metastasis of gastric cancer through GPR37 by enhancing adhesion ability. Moreover, REG4 triggers a positive feedback regulatory circuit, transducing through GPR37, ADAM17, TGF-alpha, EGFR, ERK, SP1 and finally back to REG4.

Our results from tissue array showed that REG4 is significantly over expressed in gastric cancer tissues, confirming previous studies on REG4 expression in gastric cancer and colorectal cancer [24–30]. We also proved that REG4 high expression is related to advanced stage and poor survival prognosis. Unfortunately, there was no sample available for us to test REG4 expression in peritoneal metastasis sites, nor were a good number of peritoneal metastasis patients included in the tissue array, because peritoneal metastasis patients were no longer suitable candidates for curative surgery.

Our results showed that REG4 was more abundantly expressed in gastric cancer cells and its level varied among different cell lines. High expressed cell lines, such as SNU-16, derive from ascites and might have a high tendency towards peritoneal metastasis [31], while low expressed cell lines, like MKN-45 and MKN-28, come from liver or lymph node metastasis and are likely to metastasize through a different mechanism. Such observation corroborates that high expression of REG4 contributes to peritoneal metastasis of gastric cancer [32].

REG4 is a small secretive molecule, belonging to group VII C-type lectin family. However, there is now evidence enough to prove that REG4 can also bind to polysaccharides, mannan, and heparin in the absence of calcium, mediated by two calcium independent binding sites [33]. This may contribute to the fact that, unlike other members of REG family, REG4 is responsive to some growth factors instead of inflammatory factors [12]. We showed that SP1, upon activated by TGF-alphaor EGF(data not shown), is able to induce REG4 expression by binding to an enhancer. Since we still found a considerable luciferase activity after mutation of this particular enhancer, there must be other SP1 binding sites or transcription factors downstream EGFR that participate in REG4 transcription. Nevertheless, mutating this enhancer alone was able to remarkably abrogate REG4 inducement upon TGF-alpha stimulation, which suggests it must be an important enhancer with quite powerful effect on REG4 transcription.

The receptor of REG4 is still unknown, nor its interactive partner, even though REG4 itself was isolated more than a decade ago. Luckily, we found an interesting secretive molecule-prosaposin in the differential band. Since its receptor has been identified and is proved to increase metastatic ability of cancer cells [18, 34, 35], we postulate that these two molecules may interact to exert similar biological function. Although we successfully showed their interaction and co-localization (Figure 3C, 3D, Supplementary Figure 3A, 3B), and proved that GPR37 mediated REG4′s pro-metastatic ability, we still haven't got solid evidence of their direct interaction, neither have we identified their essential binding structure. As a result, it is too bold, at this stage, to claim GPR37 is the very receptor of REG4, therefore we only recognize GPR37 as an interactive partner, which mediates pro-metastatic ability. However, given the promising data this project has demonstrated, we will continue to investigate it in the future study.

ADAM17 per se has a full-length immature form and a mature active form (whose pro-domain is cleaved). The proportion of mature ADAM17 has been showed to be responsible for its activity [23, 36], but we did not find a change in mature ADAM17 consistent with the increased TGF-alpha concentration after rhREG4 stimulation. However, there is also evident that ADAM17 phosphorylation can increase its cleavage ability [13], moreover, since ADAM maturation occurs intracellularly in the secretory pathway [22], and GPCRs recruit many kinases such as SRC, PI3-K, it may be a more straightforward way to phosphorylate ADAM17 after GPCR activation. There are relatively few study focused on the downstream mechanism of GPR37, so we have very little idea of the precise pathway which leads to ADAM17 phosphorylation at this moment. However, given that ADAM17 possesses a PDK1 specific docking site, it is plausible to postulate a pathway involving some second messenger and kinases, which promotes PDK1 translocation to the membrane and consequently activates ADAM17.

Meanwhile, since we found a significant enhance in adhesive ability, 30 potential adhesive molecules were tested in cells transfected with REG4 or control plasmid, and 6 promising molecules were found which showed significantly differential expression (≥ 3 folds) between the groups (Table 2). Among them integrin alpha 4 (ITGA4), ITGA10, ITGAX, integrin beta 4 (ITGB4) and ITGB6 which are receptors of extracellular matrix, were dramatically increased in REG4 overexpressed cells, on the contrary, desmocollin 2 (DSC2), which mediates intercellular desmosome junction, was remarkably decreased. We believe it is possible that GPR37, being a G-protein coupled receptor, connects with integrins to mediate the enhancing adhesion ability [37]. Our group will continue to investigate these hypotheses in the future.

**Table 2:** PCR array results

Molecule Name	Fold Change (Reg4/Vector)
ITGA1	1.07
ITGA2	2.59
ITGA3	1.61
ITGA4	6.04
ITGA5	1.49
ITGA6	1.59
ITGA9	2.36
ITGA10	4.01
ITGAAv	1.30
ITGAAX	3.44
ITGAAM	2.74
ITGB1	1.06
ITGB2	1.12
ITGB3	1.88
ITGB4	4.52
ITGB5	1.54
ITGB6	5.26
ITGB8	1.25
Id1	2.34
Id3	1.29
CD44	−1.12
VCAM-1	2.57
THBS1	1.90
THBS4	1.22
THBS3	1.34
THBS2	1.28
MCAM	−1.55
DSC2	−7.42
SDC4	−1.40
CDH1	−1.08

ITGA, Integrin alpha; ITGB, Integrin beta; Id, DNA-binding protein inhibitor ID; VCAM-1, Vascular cell adhesion protein 1; THMS, Thrombospondin; MCAM, Cell surface glycoprotein MUC; DSC2, Desmocollin-2; SDC4, Syndecan-4; CDH1, Cadherin-1

In conclusion, we show that REG4 promotes peritoneal metastasis through GPR37 by increasing adhesion ability. The positive feedback loop, triggered by REG4, may transfer a transient signal in to a stable one, to which drug resistance and aggressive progress may be imputed. Since the loop consists of many renowned oncogenes, we believe it plausible to formulate combinative targets to diagnose and treat peritoneal metastasis of gastric cancer in the future. Besides, circuits of such a rapid and persistent property seem a result of cancer cells counteracting human attack, and should be common among different cancer types, so it might be helpful to investigators of other tumors as well.

## MATERIALS AND METHODS

### Tissues and cell lines

Gastric tumor and adjacent non-tumorous tissues were obtained from patients under curative surgery at Shanghai Ruijin Hospital (Details in Table 1). None of the patients had received radiotherapy or chemotherapy before surgery. Clinicopathological data were collected and pathological tumor staging was determined according to the UICC TNM classification. The human immortalized gastric epithelial cell and human gastric cancer cell lines were purchased from Shanghai Institutes for Biological Sciences, Chinese Academy of Sciences.

### Cell cultures and treatment

Cells were cultured under regular conditions. For growth factor stimulation, gastric cancer cells were exposed to rhTGF-alpha (Peprotech), rhEGF (SAB), rhREG4 (Abcam) at indicated concentrations for indicated periods of time. Inhibitors were used as follows: PD98059 (Selleck) 10uM for MEK, AG-1478 (Selleck) 10uM for EGFR.

### Establishment of stable transfectants

Gastric cancer cells were stably transfected with REG4-Flag pcDNA3.1 and Flag pcDNA3.1. After 24h, and every 48h thereafter for 4 weeks, culture medium was replaced with fresh medium culture medium containing 800μg/ml of G418.

### siRNA preparation and transfection of gastric cancer cells

Gastric cancer cells were transfected with siRNAs according to the instruction manual. The sense and anti-sense strands of siRNAs were listed in Supplementary Table S1.

### Immunohistochemistry

Immunohistochemistry (IHC) was performed according to standard LSAB protocol (Dako, USA), using rabbit polyclonal antibodies against REG4 (R&D, USA).

### Total RNA isolation and quantitative realtime polymerase chain reaction

Total RNA and qRT-PCR were performed as previously described [38]. Oligonucleotide sequence of qRT-PCR primers were listed in Supplementary Table S2.

### Western blot

Protein extracts were resolved through 8%~15% SDS-PAGE, transferred to PVDF membranes, and probed with antibodies against human REG4 (R&D), EGFR(CST, USA), p-EGFR(GeneTex, USA), ERK(CST, USA), p-ERK(CST, USA), SP1(CST, USA), p-SP1(GeneTex, USA), GPR37(Abcam, USA), ADAM17(Abcam, USA), p-ADAM17(Abcam, USA) and GAPDH (Abcam, USA). Peroxidase-conjugated anti-mouse or rabbit IgG (CST, USA) was used as secondary antibody, and the antigen-antibody reaction was visualized by enhanced chemiluminescence assay (ECL, Thermo).

### Enzyme-linked immunosorbent assay (ELISA)

TGF-alpha levels in the supernatants of cultured cells were measured by a sandwich enzyme-linked immunosorbent assay (ELISA) using a commercially available kit (RayBio), as described by the manufacturer.

### Immunofluorescence analysis

For immunofluorescent staining, the cells were incubated with primary antibodies against REG4 (R&D) and GPR37 (Abcam), followed by incubation with iFluor 594 anti-rabbit IgG antibody (AAT Bioquest) or Alexa488-conjugated anti-goat IgG antibody (Life technologies). For confocal microscopy, the cells on cover-slips were counterstained with DAPI and imaged using a confocal laser-scanning microscope (Carl Zeiss) with a core data acquisition system (Applied Precision).

### Affinity purification of REG4-Interacting proteins and immunoprecipitation

The whole cell extracts of SGC-7901/vector-Flag and SGC-7901/REG4-Flag cells were immunoprecipitated with anti-Flag M2 affinity gel (Sigma) according to manufacturer's instruction. The immunoprecipitates were resolved on an SDS-PAGE denaturing gel, visualized by Coomassie blue, and the protein band of interest was removed for mass spectrometric analysis. For Co-IP, immunoprecipitates were used to continue Western blot.

### Luciferase reporter assay

Luciferase reporter assay was performed as previously described [39].

### Chromatin immunoprecipitation assay

Chromatin immunoprecipitation assay was performed using a ChIP kit (Millipore) according to the manufacturer's instruction. Primers designed to target the REG4 promoter region spanning the site of interaction with SP1 were listed in Supplementary Table S2.

### Adhesion assay

Adhesion of gastric cancer cells to different components of ECM was evaluated using a kit (CBA) according to the manufacturer's instruction. Adhesion to Matrigel and murine peritoneum were performed on culture plates that were pre-coated with Matrigel and murine peritoneum and were pre-treated with 10% BSA to block nonspecific binding proteins. Cells were then suspended in serum-free medium at 37°C, and were allowed to adhere to the plate bottom for 30 min. After removal of the non-adherent cells by gently washing with PBS for three times, the adhered cells were quantified by MTT or counted as cells per field of view under fluorescent microscopy.

### Peritoneal metastasis model

Four-week-old male BALB/C nude mice were purchased from the Institute of Zoology, Chinese Academy of Sciences of Shanghai. All experiments were performed in accordance with the official recommendations of the Chinese animal community. Cells were resuspended in 0.1 ml PBS and injected into the abdominal cavity. The mice were sacrificed after 10 weeks, and the volume of ascites and quantity of nodules present in the abdominal cavity of each mouse were measured.

### Ethics statement

All animal experiments were conducted according to China guidelines for animal experimentation and approved by the Institutional Animal Care Committee of our hospital. All patients provided written informed consent before enrollment and the Ethic Committee of Ruijin Hospital approved the study protocol. The study was carried out according to the provisions of the Helsinki Declaration of 1975.

### Statistical methods

Statistical analyses were performed using Kaplan–Meier method, Cox proportional hazards regression, Student's t-test, one-way ANOVA, chi-squared test, and Fisher's exact test. Differences were considered statistically significant in a two-tailed test for p-values <0.05. The statistical analysis software used was SPSS Version 22.0.

## SUPPLEMENTARY FIGURES AND TABLES


