# Supplementary material for: REG4 promotes peritoneal metastasis of gastric cancer through GPR37
Source: Oncotarget. 2016 Mar 28;7(19):27874–88. doi: 10.18632/oncotarget.8442 (PMC5053694; doi:10.18632/oncotarget.8442)
Supplement: Supplementary file 1 [file oncotarget-07-27874-s001.pdf]

SUPPLEMENTARY FIGURES AND TABLES

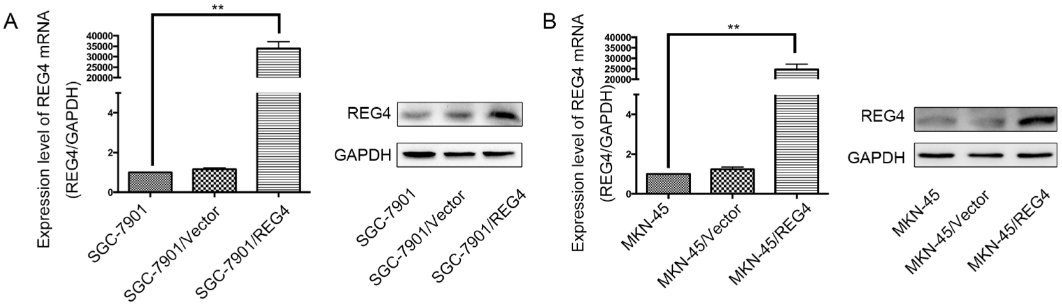

Supplementary Figure S1: mRNA and protein level of REG4 in SGC-7901/REG4 A. and MKN-45/REG4 B. compared with control cells.

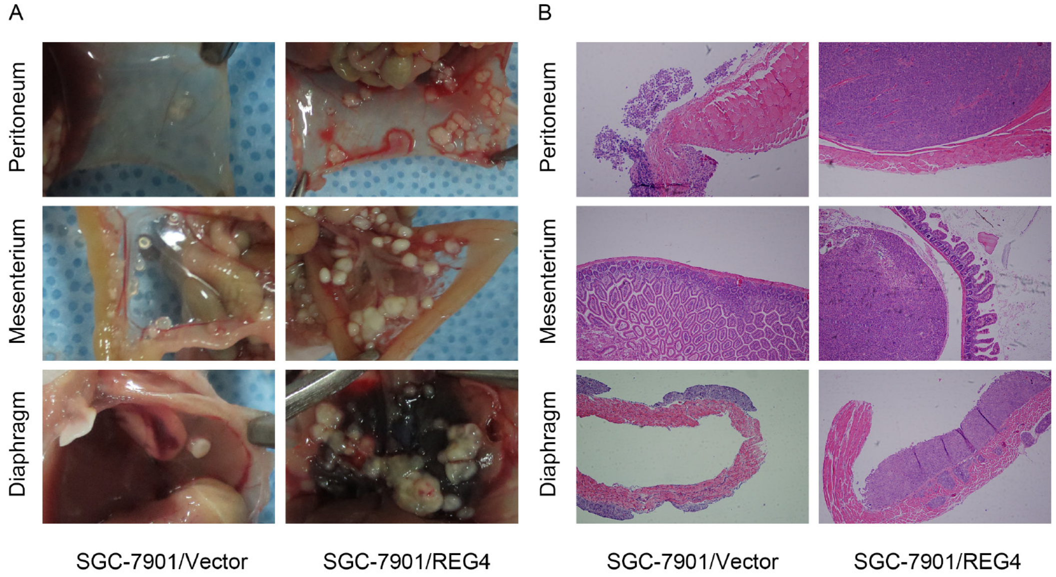

Supplementary Figure S2: REG4 promotes peritoneal dissemination of GC cells in vivo. A. Representative photos of tumor formation at the mesentery, diaphragm and peritoneum are shown. **B.** The histological appearance of disseminated tumors in the abdominal cavities as indicated was assayed by H & E staining.

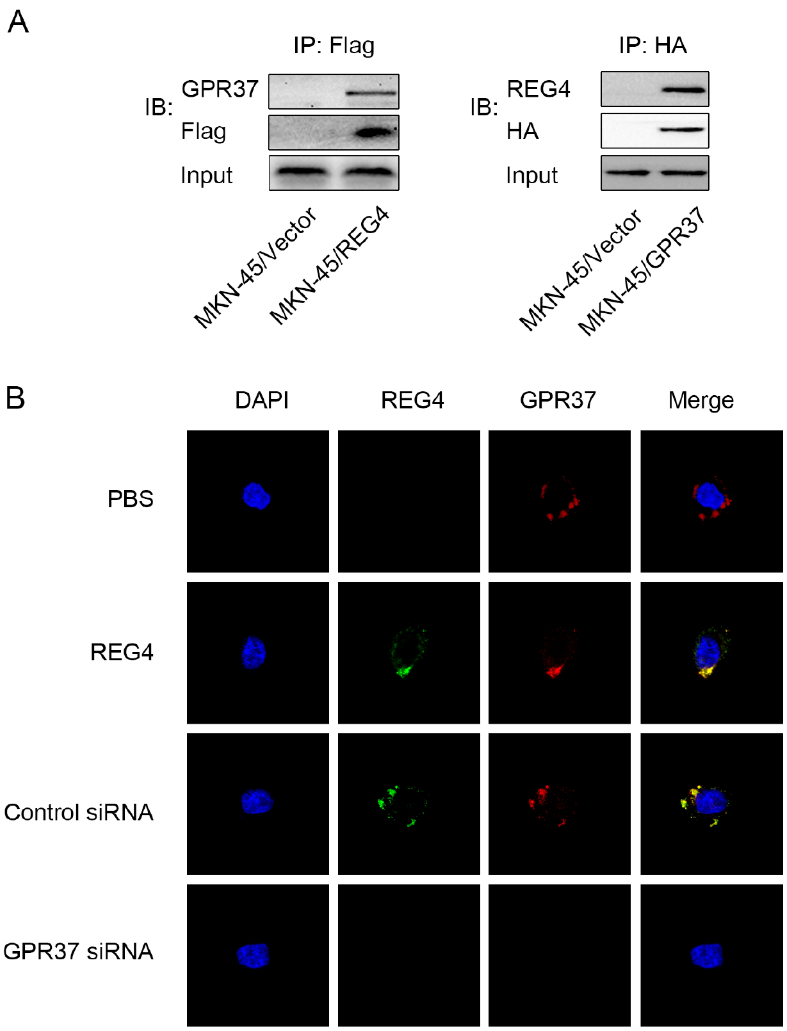

Supplementary Figure S3: GPR37 is in the same complex with REG4. A. Immunoprecipitation of the whole cell extracts from MKN-45/REG4-Flag with anti-Flag antibody, and from MKN-45/GPR37-HA with anti-HA antibody. **B.** Confocal microscopy for MKN-45 cells stained with anti-REG4, anti-GPR37 antibody and iFluor 594 anti-rabbit IgG, Alexa 488 anti-goat IgG second antibody. The cells were treated with PBS (row 1) or recombinant REG4 (rows 2-4), and were untransfected (row 2), transfected with control-siRNA (row 3), or GPR37-siRNA (rows4). Cell nuclei were counterstained with DAPI.

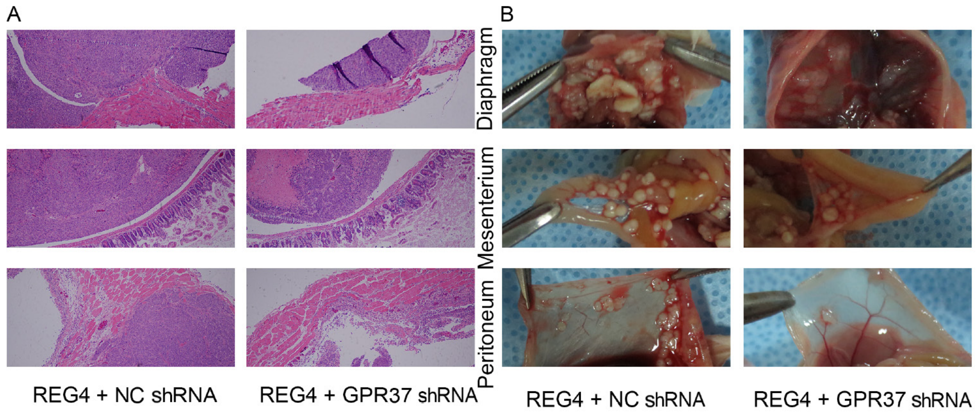

Supplementary Figure S4: Knocking down GPR37 abrogates the pro-peritoneal metastasis effect of REG4. A. The histological appearance of disseminated tumors in the abdominal cavities as indicated was assayed by H & E staining. **B.** Representative photos of tumor formation at the mesentery, diaphragm and peritoneum are shown.

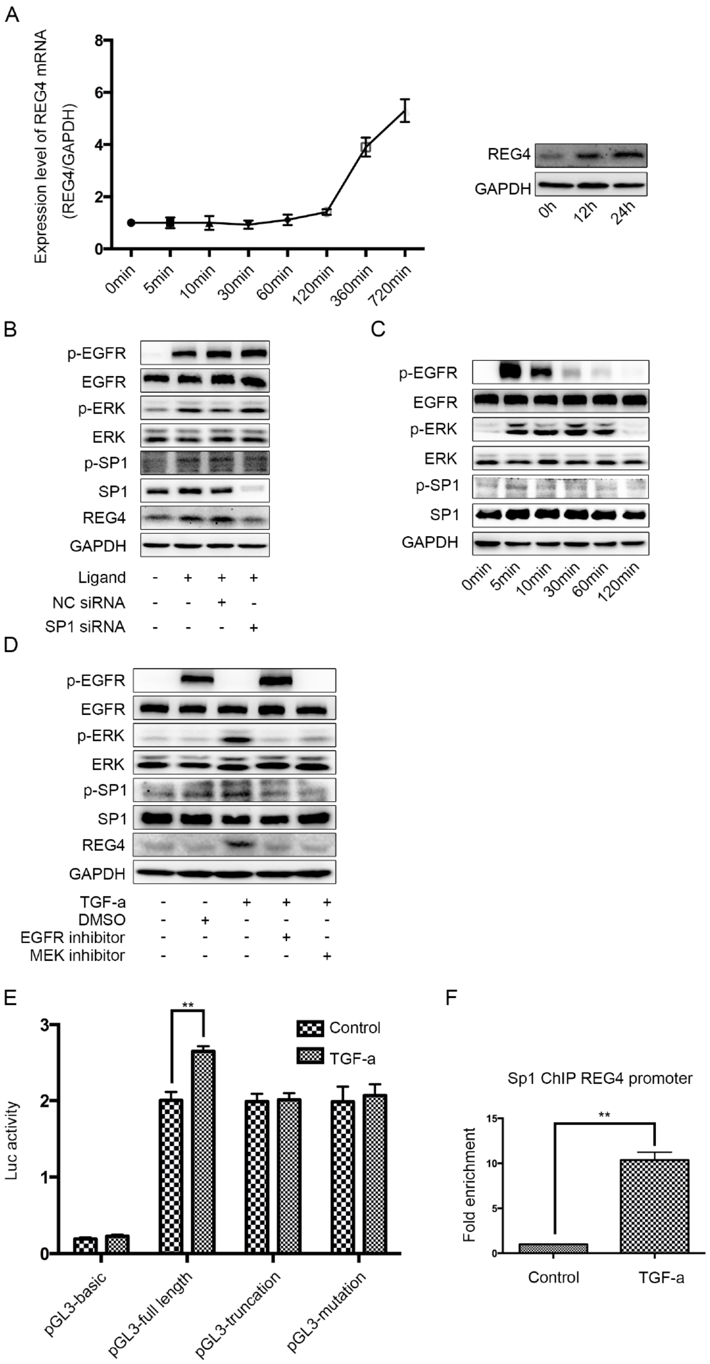

Supplementary Figure S5: TGF-alpha induces REG4 expression through EGFR pathway in MKN-45. A. TGF-alpha (10nM) increases REG4 on both mRNA and protein level. **B.** Knocking down SP1 abrogates REG4 inducement upon TGF-alpha stimulation. **C.** Phosphorylation level of EGFR, ERK, SP1 were analyzed after TGF-alpha (10nM) stimulation. **B, D.** Activation of EGFR pathway and expression of REG4 were tested with or without EGFR inhibitor, MEK inhibitor and SP1 siRNA, respectively, after TGF-alpha (10nM) stimulation. **E.** Luciferase activity of a reporter construct harboring different REG4 promoter with or without TGF-alpha (10nM) stimulation. **F.** SP1 occupancy (fold enrichment) on REG4 promoter areas with or without TGF-alpha (10 nM) stimulation. Error bars correspond to mean ± SD of at least three independent experiments. *P < 0.05, **P<0.01.

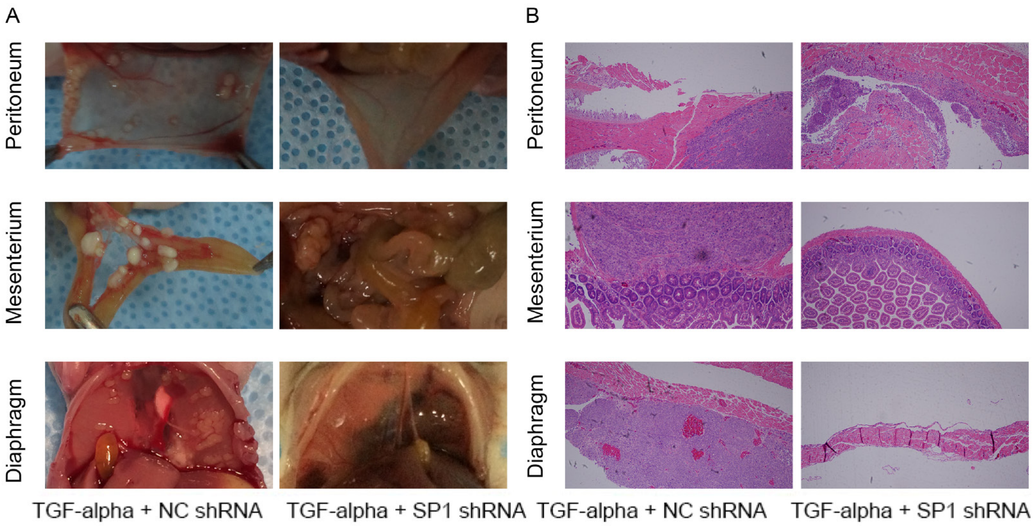

Supplementary Figure S6: Knocking down SP1 abrogates the pro-peritoneal metastasis effect of REG4. A. Representative photos of tumor formation at the mesentery, diaphragm and peritoneum are shown. **B.** The histological appearance of disseminated tumors in the abdominal cavities as indicated was assayed by H & E staining.

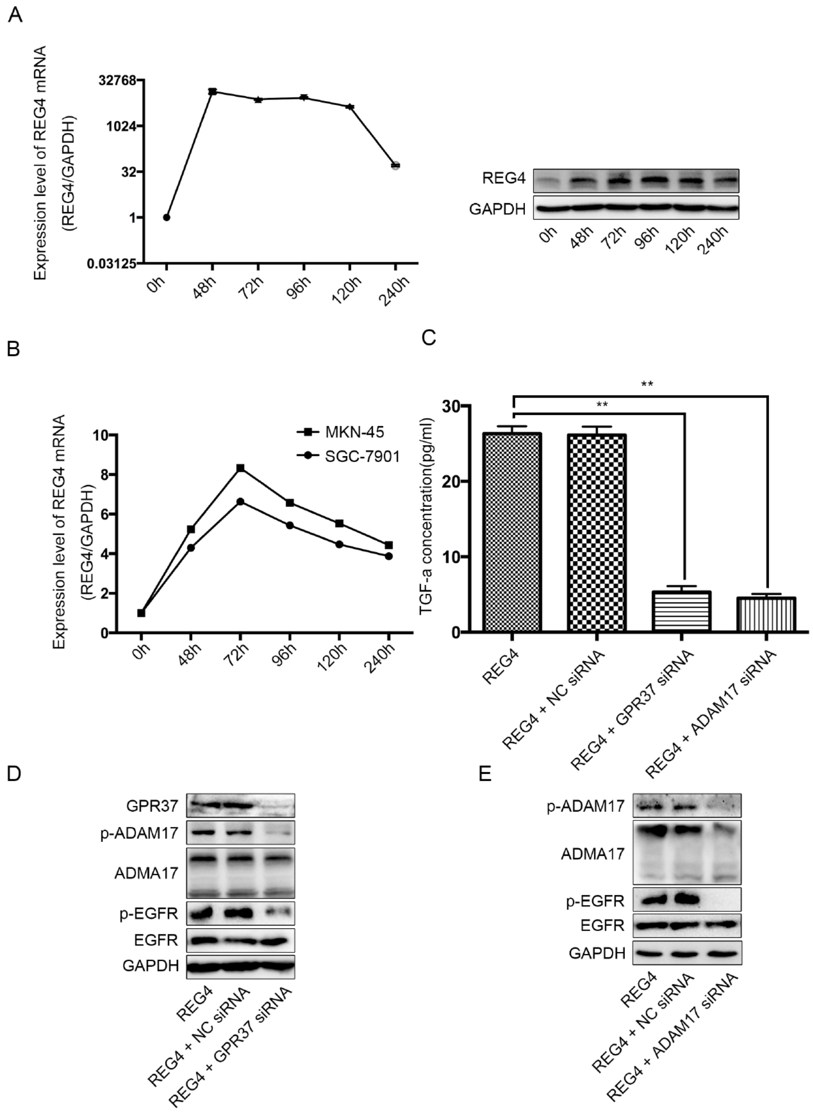

Supplementary Figure S7: REG4 triggers a positive feedback loop. A. mRNA and protein level after transient transfection of REG4 plasmid in MKN-45. **B.** mRNA level after REG4 stimulation (10ng/ml) in SGC-7901 and MKN-45. **C.** TGF-alpha concentration in medium supernatant with or without GPR37 siRNA or ADAM17 siRNA after rhREG4 (10ng/ml) stimulation in MKN-45. **D, E.** Phosphorylation and total level of ADAM17 and EGFR with or without GPR37 siRNA or ADAM17 siRNA after rhREG4 (10ng/ml) stimulation in MKN-45.

Supplementary Table S1: The sense and anti-sense strands of siRNAs

siRNA	sense	antisense
SP1 siRNA-1	5′-CCAACAGAUUAUCACAAUdTdT-3′	3′-dTdTGGUUGUCUAAUAGUGUUUA-5′
SP1 siRNA-2	5′-GGCUGGUGGUGAUGGAAUAdTdT-3′	3′-dTdTCCGACCACCACUACCUUAU-5′
ADMA17 siRNA-1	5′-GCUUGAUUCUUUGCUCUCAdTdT-3′	3′-dTdTCGAACUAAGAAACGAGAGU-5′
ADAM17 siRNA-2	5′-GCUUGUUCAUCGAGUGAAAdTdT-3′	3′-dTdTCGAACAAGUAGCUCACUUU-5′
GPR37 siRNA-1	5′-CGUACAGAUGUACUACGAAdTdT-3′	3′-dTdTGCAUGUCUACAUGAUGCUU-5′
GPR37 siRNA-2	5′-GGACCGGGAAAUUCUGCAAdTdT-3′	3′-dTdTCCUGGCCCUUUAAGACGUU-5′

Supplementary Table S2: Oligonucleotide sequence of qRT-PCR primers

Gene	Forward primer	Reverse primer
Reg4	5′-TGGAGCAGCAACGAATGC-3′	5′-TGCTCTATGGTCGGTACTTGCA-3′
ChIP(Reg4)	5′-GAGGATGCAAAGGCACAAGAACG-3′	5′-TTTGGGGCACCCATCATCCA-3′
SP1	5′-GAGCAAAACCAGCAGACACA-3′	5′-ACTGTTGGTGTCCGGATGAT-3′
GAPDH	5′-GGACCTGACCTGCCGTCTAG-3′	5′- GTAGCCCAGGATGCCCTTGA-3′
